# Whole-body magnetic resonance angiography for the assessment of extent and severity of extra-cardiac atherosclerosis in patients with newly diagnosed coronary artery disease

**DOI:** 10.1186/1532-429X-11-S1-P273

**Published:** 2009-01-28

**Authors:** Stephanie Lehrke, Benjamin Egenlauf, Henning Steen, Constanze Merten, Dirk Lossnitzer, Evangelos Giannitsis, Hugo A Katus

**Affiliations:** Medizinische Universitätsklinik Heidelberg, Heidelberg, Germany

**Keywords:** Coronary Artery Disease, Coronary Angiography, Risk Score, Magnetic Resonance Angiography, Vessel Segment

## Introduction

The presence of extra- cardiac atherosclerosis is associated with an adverse prognosis in patients with coronary artery disease (CAD).

## Purpose

We hypothesized that whole body magnetic resonance angiography (MRA) is a valuable tool for the non-invasive assessment of the severity of systemic atherosclerosis in patients (pts) with CAD. We further propose the use of an atherosclerosis score index as a novel marker for the severity of systemic atherosclerotic burden.

## Methods

Whole-body MRA was performed on a 1.5 T clinical scanner equipped with a moving table top extender (Intera Achieva^®^, Philips Medical Systems) in 31 pts scheduled to undergo coronary angiography for suspected CAD. None of the pts had known extra-cardiac arterial disease. The arterial tree was divided into 40 segments from the carotid arteries to the arteries of the lower leg which were graded with regard to their luminal narrowing (≤10%, 11–25%, 26–50%, 51–75%, 76–90%, 91–99%, occlusion). All segments received a score according to the severity of stenosis, ranging from 1 for a normal vessel to 8 for a total occlusion. The atherosclerosis score index (ASI) for each patient was calculated by dividing the sum of vessel scores by the number of analysed segments.

## Results

Whole-body MRA was obtained in good quality from all pts and a total of 1088 arterial segments were analysed. Coronary angiography revealed significant CAD in 19/31 pts. These pts had a higher atherosclerosis score index (1.77 ± 0.11 vs 1.46 ± 0.06, *p* = 0.03) and exhibited more vessel segments with a significant (>25%) stenosis (2 ± 0.48 vs 0.83 ± 0.34, *p* = 0.05). ASI showed a good correlation with PROCAM (R = 0.49, p = 0.01) and Framingham (R = 0.4, p = 0.04) risk scores.

## Conclusion

Our findings demonstrate that pts with newly diagnosed CAD exhibit a higher systemic atherosclerotic burden compared to pts in whom CAD was ruled out by coronary angiography. We are the first to describe the use of a whole body atherosclerosis score index as marker for systemic atherosclerotic burden which correlates well with established risk scores. This novel technique may aid in the risk-stratification in pts with suspected CAD and may aid in the evaluation of new therapies that target atherosclerotic disease.

